# Streptozocin/5-fluorouracil chemotherapy of pancreatic neuroendocrine tumours in the era of targeted therapy

**DOI:** 10.1007/s12020-021-02859-y

**Published:** 2021-09-04

**Authors:** Harald Lahner, Annie Mathew, Anna Lisa Klocker, Nicole Unger, Jens Theysohn, Jan Rekowski, Karl-Heinz Jöckel, Sarah Theurer, Kurt Werner Schmid, Ken Herrmann, Dagmar Führer

**Affiliations:** 1 Department of Endocrinology, Diabetes and Metabolism and Division of Laboratory Research, Endocrine Tumor Center at WTZ/ Comprehensive Cancer Center and ENETS Center of Excellence, University of Duisburg-Essen, Essen, Germany; 2 Institute of Diagnostic and Interventional Radiology and Neuroradiology, University of Duisburg-Essen, Essen, Germany; 3 Institute of Medical Informatics, Biometry, and Epidemiology, University of Duisburg-Essen, Essen, Germany; 4 Institute of Pathology, University of Duisburg-Essen, Essen, Germany; 5 Department of Nuclear Medicine, University of Duisburg-Essen, Essen, Germany

**Keywords:** Streptozocin, 5-fluorouracil, Pancreatic neuroendocrine tumor, Objective response, Survival

## Abstract

**Purpose:**

The role of streptozocin-based chemotherapy (STZ CTx) in advanced, well-differentiated pancreatic neuroendocrine tumours (PanNET) and the best sequence of treatments in advanced PanNET are unclear. We examined the outcomes after STZ CTx in patients who had been selected according to the current therapeutic guidelines.

**Methods:**

Data from 50 PanNET patients consecutively treated with STZ CTx between 2010 and 2018 were analysed. The endpoints of the study were the objective-response rate (ORR), progression-free survival (PFS), and overall survival (OS).

**Results:**

STZ CTx was the first-line treatment in 54% of patients. The PanNET grades were as follows: 6% G1, 88% G2, and 6% well-differentiated G3. The ORR was 38%. Stable disease was the best response in 38% of patients and 24% showed progressive disease. Treatment was discontinued because of toxicity in one patient. Median PFS and OS were 12 (95% confidence interval (CI), 8.5–15.5) and 38 months (95% CI, 20.4–55.6), respectively. In the Kaplan-Meier analysis, the median OS was 89 months (95% CI, 34.9–143.1) for STZ CTx as first-line therapy compared with 22 months (95% CI, 19.3–24.7; *p* = 0.001, log-rank test) for subsequent lines. Bone metastases negatively impacted survival (HR, 2.71, *p* = 0.009, univariate analysis, HR, 2.64, *p* = 0.015, multivariate analysis, and Cox regression).

**Conclusions:**

In patients selected according to current guidelines, PFS, and OS after STZ CTx were lower than previously reported, whereas ORR was unchanged. First-line treatment was positively associated with OS and the presence of bone metastases was negatively associated with OS. Pre-treatment with targeted or peptide-receptor radionuclide therapy did not alter ORR, PFS, or OS.

## Introduction

Pancreatic neuroendocrine tumours (PanNET) are rare neoplasms with an annual incidence of 0.48/100,000 [1]. Surgical removal is the only curative therapy. At presentation, 60%–80% of patients have unresectable disease due to local extension or metastases. Hence, only palliative interventions can be offered [2]. Streptozocin-based chemotherapy (STZ CTx) has been an established first-line treatment since 1980. Initially, response rates (RR) exceeding 60% and a sustained median progression-free survival (PFS) of 36 months were reported [3]. Subsequent series found very heterogeneous results with RR between 6% and 55% and PFS of 4–23 months [4]. These conflicting results are attributed to heterogeneous patient cohorts and classification systems.

In 2010, a unified PanNET assessment was established and novel treatment options have been developed since then. The World Health Organisation (WHO) introduced a new classification system that abolished the distinction between well-differentiated endocrine tumours of benign behaviour and endocrine carcinomas, highlighting the malignant potential of all neuroendocrine tumours (NET) based on the proliferation marker, Ki-67 [5]. The validity of this grading system was confirmed in several studies [6].

Sunitinib, a multikinase inhibitor, approved by the European medicines agency (EMA) in 2010 and the food and drug administration (FDA) in 2011, was the first licensed therapeutic alternative to STZ CTx for progressive, well-differentiated PanNET. Approval of sunitinib was based on a double-blind, randomised study demonstrating an increase in PFS from 5.5 to 11.4 months [7]. In the same year, everolimus, an oral mTOR inhibitor, was also approved by the EMA and FDA for the treatment of progressive, well-differentiated PanNET. Approval of everolimus was also based on double-blind, randomised data [8]; PFS increased from 4.6 to 11.0 months in this study.

Since their approval, sunitinib and everolimus have competed with STZ CTx in the treatment of well-differentiated PanNET. However, the importance of the individual substances within the therapy algorithm has not been established because of the lack of comparative studies. Furthermore, peptide receptor radionuclide therapy (PRRT) is commonly used in PanNET, but whether prior PRRT influences the outcome of STZ-CTx is currently unknown [9, 10]. In the current guidelines, STZ CTx is one of the standard therapies [11–13]. The length of practical experience supports this approach. However, there are no studies in which the current therapeutic alternatives have been available. Tumour classification, therapeutic thresholds, and alternatives differ considerably in published studies [4, 14–17]. In addition, grading based on Ki-67 has only been established as a mandatory part of NET baseline classification since 2010.

The aims of the present study were thus (1) to determine the outcome of STZ CTx in a well-defined patient population, treated according to current guidelines, in which the current therapeutic alternatives are available; (2) to assess the influence of previous targeted therapy and PRRT on objective response rate (ORR), PFS, and overall survival (OS), and (3) to detect factors influencing therapeutic outcomes.

## Materials and methods

### Patients

Patients with histologically confirmed, well-differentiated and locally advanced or metastatic PanNET, who received STZ CTx between January 2010 and January 2018, were identified from our prospective database at the European Neuroendocrine Tumour Society (ENETS) Centre of Excellence at the University Hospital of Essen. The follow-up period was extended until April 2020. Patients with hereditary tumours (multiple endocrine neoplasia type 1 or von Hippel–Lindau disease) were excluded. To ensure consistency, indication for therapies was determined according to ENETS guidelines by an experienced, multidisciplinary tumour board (MTB) [11, 12]. All therapies were administered in-house at our centre.

### Chemotherapy

The STZ CTx consisted of 500 mg/m² of streptozocin in 100 ml of 0.9% NaCl IV infusion given over 30 min, followed after 1 h by 400 mg/m² of fluorouracil (5-FU) infusion in 100 ml of 0.9% NaCl given over 30 min. Adequate peri-interventional hydration was ensured by administering at least 1000 ml of 0.9% NaCl IV infusion. A 5-hydroxytryptamine (5-HT3) antagonist was administered 30 min before the start of therapy. Dexamethasone (8 mg) was administered per os at the beginning of each chemotherapy day. The therapy was implemented over five consecutive days, with a cycle length of 42 days. In case of impaired performance status or toxicity, a delay of up to 2 weeks was provided. The first staging was performed using computed tomography (CT) after three cycles. Patients who did not show progression received the intended number of six cycles, unless unacceptable toxicity occurred.

### Follow-up and evaluation of tumour response

A baseline CT scan was performed within 4 weeks before starting STZ CTx. After three completed treatment courses, the first evaluation of therapeutic response (history, physical examination, CT or MRI scan, and laboratory investigations) was scheduled. In case of stable disease (SD) or remission, STZ CTx was continued until the planned number of six cycles. Within 4 weeks after the last cycle and at 3-month intervals, follow-up examinations (CT or MRI) were performed. Hybrid imaging (^68^Ga DOTATOC positron emission tomography (PET)/CT) was included at the initial presentation and at least every 12 months within the surveillance schedule. After 1 year of SD, partial remission (PR) or complete remission (CR), follow-up intervals were extended from 3 to a maximum of 6 months, according to the MTB decision. Response to treatment was evaluated using the international criteria proposed by the Response evaluation criteria in solid tumours (RECIST) committee. At each scheduled time point, chromogranin A (CgA), hematologic, renal, hepatic, endocrine, and coagulation parameters were measured and clinical symptoms were recorded according to common terminology criteria for adverse events (CTCAE), version 3.0.

### Pathology of the tumours

The presence of PanNET was confirmed morphologically and immunohistochemically in all patients. The Ki-67 index was indicated using the MIB-1 antibody, taking into consideration the area of highest activity. Tumour grading was performed according to the WHO/ENETS criteria [18, 19]. Low-grade (G1) PanNET were defined as tumours having a Ki-67 index of ≤2% and intermediate-grade (G2) PanNET was defined as tumours having a Ki-67 index between 3% and 20%. Three patients had well-differentiated, high proliferative PanNET with a Ki-67 index of >20%. The analyses were performed by one pathologist with expertise in endocrine and pancreatic tumours. The pathologist was blinded to the patients’ history.

### Statistical methods

Response and tumour characteristics were compared using Fisher’s exact test. PFS was recorded as the time between the start of treatment and radiological progression (based on RECIST 1.1) or death. Survival rates were calculated using the Kaplan-Meier method. OS from diagnosis was defined as the time between PanNET diagnosis and death or the last follow-up. OS from the start of chemotherapy was defined as the time between the start of treatment and death or the last follow-up. Univariate and multivariate Cox regression analyses were performed to identify prognostic factors. Statistical differences in PFS and OS between patient groups were estimated using the log-rank test. A *p*-value of <0.05 was considered significant. All statistical calculations were performed using IBM SPSS Statistics for Windows, Version 25.0 (IBM Corp., Armonk, NY).

## Results

### General characteristics

The cohort consisted of 50 consecutive PanNET patients with well-differentiated morphology. All patients were accessible for analysis (Table 1). Forty-one tumours (82%) were non-functioning and nine tumours (18%) were functioning. Three tumours were graded as G1 and the majority (88%) were G2 neoplasms, according to the WHO 2017 classification. Three patients had well-differentiated tumours, with elevated Ki-67-proliferation rates of 25% (*n* = 2) and 30% (*n* = 1), corresponding to NET G3. Stage-IV disease was present in 96% of patients and the majority of these patients had liver metastases. Distant organs (≥2) were involved in 50% of patients.

**Table 1:** Patient characteristics

	*n* (%)
Sex	
Male	26 (52)
Female	24 (48)
Age at treatment start (years)	61 (28–82)
Disease duration at treatment start (months)	6.5 (1–159)
Functionality	
Non-functioning	41 (82)
Gastrinoma	4 (8)
Insulinoma	3 (6)
VIPoma	1 (2)
PTHrP producing	1 (2)
Tumour grade (WHO 2017)	
G1	3 (6)
G2	44 (88)
G3	3 (6)
Ki-67 index (%)	
≤5%	17 (34)
>5%–10%	13 (26)
>10%–20%	17 (34)
>20%–30%	3 (6)
Well-differentiated morphology	50 (100)
Organ tumour involvement	
No distant disease site	2 (4)
1 distant disease site	23 (46)
2 distant disease sites	15 (30)
≥3 distant disease sites	10 (20)
Metastases	
Lymph node involvement only	2 (4)
Liver metastases	48 (96)
Distant metastases other than liver	25 (50)
Bone metastases	19 (38)
Treatment line	
1st line	27 (54)
2nd line	13 (26)
>2nd line	10 (20)
Prior treatment	
None	27 (54)
Somatostatin analogues	16 (32)
Sunitinib or everolimus	7 (14)
PRRT	13 (26)
Interferon	1 (2)
Other CTx (Tem/Cap^a^, Carbo/Eto^b^)	4 (8)
Previous resection of primary tumour	23 (46)
ECOG PS at STZ CTx start	
0	19 (38)
1	24 (48)
2	7 (14)
Baseline status	
Radiologically progressive ≤12 months	33 (66)
Clinically progressive ≤6 months	17 (34)

Age and disease duration at treatment start are given as median (range), categorical parameters as absolute and relative frequencies (*n* = 50)

^a^Tem/Cap temozolomide/ capecitabine, ^b^Carbo/Eto carboplatin/ etoposide

The median age at the start of therapy was 61 years (range, 28–82 years). Approximately half of the patients (*n* = 27, 54%) were administered STZ CTx as the first-line treatment. The median time from diagnosis to the onset of chemotherapy was 6.5 months (range, 1–159). Of those patients with prior systemic treatments (*n* = 23), the majority had received long-acting somatostatin analogs (SSA, *n* = 16) or PRRT (*n* = 13); nine of the PRRT-treated patients received combination treatment with SSA. Seven patients were pretreated with targeted therapy (*n* = 4 sunitinib, *n* = 3 everolimus). Four patients received other CTx (temozolomide-based or carboplatin/etoposide) and one patient received prior treatment with interferon. Twenty-three patients underwent surgery of the primary tumour (46%). At baseline, 66% of patients had radiologically proven progressive disease (PD). Another 17 subjects (34%) had clinically PD, characterized by new-onset or worsened abdominal pain (*n* = 15), nausea (*n* = 5), or ascites (*n* = 3). Four patients showed radiological signs of peritoneal carcinomatosis.

### Objective response

CR and PR as best responses were observed in 1 (2%) and 18 (36%) patients, respectively, corresponding to an ORR of 38%. SD as the best response, including mixed response in one case, was documented in 19 cases (38%), accounting for an overall disease control rate (DCR; CR + PR + SD) of 76%. PD was noted in 24% of all cases. Among the functional syndromes, the ORR was 22.2% (2/9), and the DCR was 77.8% (7/9). The four patients with gastrinomas each had SD and PD twice, and the three subjects with insulinomas showed PR once and SD twice. At PTHrPoma PR and at VIPoma SD was the best response. In 47 patients, the complete biochemical course of the tumour marker CgA was evaluated before, during and after STZ CTx. A decrease of CgA levels of more than 30% during therapy was associated with a significantly superior ORR (69% vs. 23%, *p* = 0.004, Fisher’s exact test). Prior targeted therapy, previous PRRT, SSA, Ki-67 index, number of distant metastases, or the progression status at baseline did not significantly affect ORR. A detailed analysis of the response to STZ CTx is summarised in Table 2.

**Table 2:** Response to STZ CTx

	Objective response No. (%)	p*	Stable disease No. (%)	Progressive disease No. (%)
All patients (*n* = 50)	19 (38.0)		19 (38.0)	12 (24.0)
**Treatment line**		*0.387*		
1st line (*n* *=* 27)	12 (44.4)		10 (37.0)	5 (18.5)
2nd or higher line (*n* *=* 23)	7 (30.4)		9 (39.1)	7 (30.4)
**Previous SSA therapy**		*>0.99*		
Yes (*n* = 16)	6 (37.5)		5 (31.3)	5 (31.3)
No (*n* = 34)	13 (38.2)		14 (41.2)	7 (20.1)
**Previous targeted therapy**		*0.229*		
Yes (*n* = 7)	1 (14.3)		3 (42.9)	3 (42.9)
No (*n* = 43)	18 (41.9)		16 (37.2)	9 (20.9)
**Previous PRRT**^a^		*0.742*		
Yes (*n* = 13)	4 (30.8)		4 (30.8)	5 (38.5)
No (*n* = 37)	15 (40.5)		15 (40.5)	7 (18.9)
**Tumour type**		*0.452*		
Functioning (*n* = 9)	2 (22.2)		5 (55.6)	2 (22.2)
Non-functioning (*n* = 41)	17 (41.5)		14 (34.2)	10 (24.4)
**Affected organ systems**		*0.244*		
≤1 (*n* = 25)	12 (48.0)		9 (36.0)	4 (16.0)
≥2 (*n* = 25)	7 (28.0)		10 (40.0)	8 (32.0)
**Bone metastases**		*0.237*		
Yes (*n* = 19)	5 (26.3)		6 (31.6)	8 (42.1)
No (*n* = 31)	14 (45.2)		13 (41.9)	4 (12.9)
**ECOG PS**^b^ **at start**		*0.695*		
≤1 (*n* = 43)	17 (39.5)		16 (37.2)	10 (23.3)
2 (*n* = 7)	2 (28.6)		3 (42.9)	2 (28.6)
**Grading**		*>0.99*		
Ki-67 ≤ 15% (*n* = 32)	12 (37.5)		13 (40.6)	7 (21.9)
Ki-67 > 15% (*n* = 18)	7 (38.9)		6 (33.3)	5 (27.8)
**Progression status at start**		*0.767*		
Radiologically (*n* = 33)	12 (36.4)		11 (33.3)	10 (30.3)
Tumour burden/symptoms (*n* = 17)	7 (41.2)		8 (47.1)	2 (11.8)
**Time from initial diagnosis**		*0.773*		
≤1 year (*n* *=* 27)	11 (40.7)		10 (37.0)	6 (22.2)
>1 year (*n* = 23)	8 (34.8)		9 (39.1)	6 (26.1)
**CgA**^c^ **decrease**		***0.004***		
0–30% (*n* = 31)	7 (22.6)		15 (48.4)	9 (29.0)
>30% (*n* = 16)	11 (68.8)		3 (18.8)	2 (12.5)

All *p*-values have been specified as italicized values and all values showing statistical significance were highlighted using bold characters

^a^*PRRT* peptide receptor radionuclide therapy, ^b^*ECOG PS* Eastern Cooperative Oncology Group performance status, ^c^*CgA* Chromogranin A

*p-value by Fisher’s exact test (objective response vs. stable disease or progression).

### Progression-free survival and overall survival

**Fig. 1:**
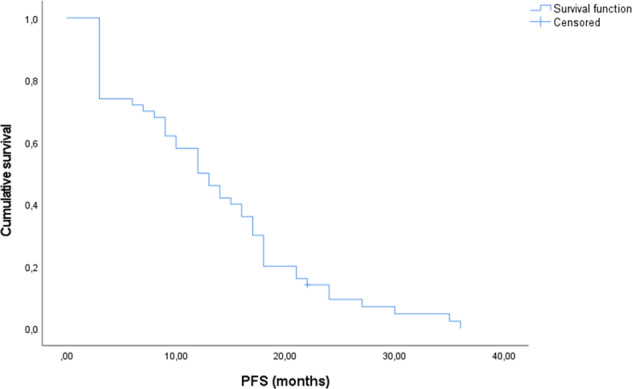
PFS, expressed in months (*n* = 50)

At the time of analysis, 49 of the 50 patients had radiologically proven PD. The median PFS after treatment with STZ CTx was 12.0 months, according to Kaplan–Meier analysis (95% confidence interval (CI), 8.5–15.5 months; Fig. 1). Four patients (8%) had a long-term response of more than 2 years. The mean previous staging interval at the time point of progression was 3.52 months (95% CI, 3.15–3.90 months).

**Fig. 2:**
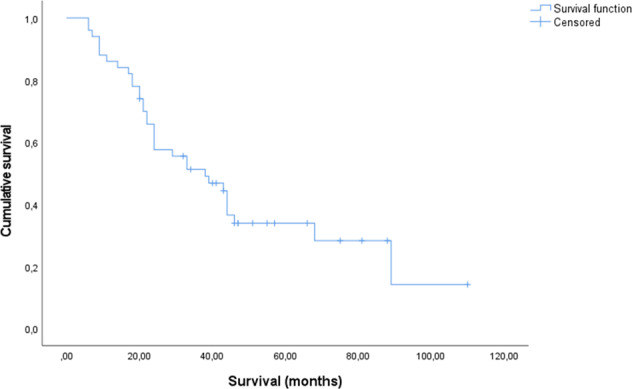
Survival from the start of treatment, expressed in months (*n* = 50)

The median survival from the start of treatment with STZ CTx was 38 months in the whole patient group (95% CI, 20.4–55.6 months). The OS rate was 57.6% (95% CI, 43.9–71.3%) at 2 years and 33.9% (95% CI, 19.8–48.0%) at 5 years, according to Kaplan-Meier analysis (Fig. 2). The overall median survival from diagnosis was 64 months (95% CI, 35.6–92.5 months). At the time of analysis, 33 cohort patients (66%) had died.

**Fig. 3:**
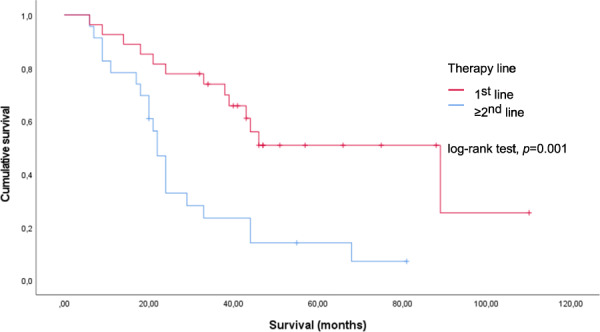
Survival from the start of treatment (months) according to therapy line (*n* = 50)

**Fig. 4:**
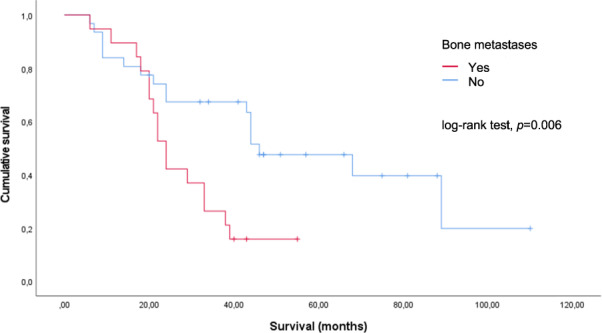
Survival from the start of treatment (months) according to bone metastases (*n* = 50)

Parameters that could affect PFS and OS are shown in Table 3. Univariate and multivariate analyses were performed using the Cox regression model. First-line treatment and the presence of bone metastases had a significant impact on survival. In Kaplan–Meier analyses, median survival from the start of treatment was 89 (95% CI, 34.9–143.1) vs. 22 (95% CI, 19.3–24.7) months in patients with first vs. later therapy lines (*p* = 0.001, log-rank test) (Fig. 3). The presence of bone metastases was associated with a shorter OS of 24 (95% CI, 20.8–27.2) vs. 46 (95% CI, 26.3–65.7) months (*p* = 0.006, log-rank test) (Fig. 4). PFS was not influenced by previous targeted therapy, PRRT, bone metastases or therapy line.

**Table 3:** Clinical parameters and their impact on PFS and OS based on univariate and multivariate analyses

Variable	Univariate			Multivariate		
	HR	95% CI	*p*-value	HR	95% CI	*p*-value
**PFS**						
Previous targeted therapy^a^ Yes (*n* = 7) vs. no (*n* = 43)	0.650	0.273–1.547	*0.330*	0.554	0.220–1,392	*0.209*
Previous PRRT Yes (*n* = 13) vs. no (*n* = 37)	1.724	0.886–3.357	*0.109*	1.919	0.956–3,855	*0.067*
Resection of primary tumour (*n* = 23) vs. no resection (*n* = 27)	0.969	0.541–1.736	*0.915*	0.985	0.524–1,849	*0.961*
Bone metastases Yes (*n* = 19) vs. no (*n* = 31)	1.144	0.625–2.093	*0.663*	1.123	0.608–2,074	*0.711*
**OS from STZ CTx**						
Previous targeted therapy^a^ Yes (*n* = 7) vs. no (*n* = 43)	1.324	0.544–3.220	*0.537*	1.464	0.543–3.946	*0.452*
Previous PRRT Yes (*n* = 13) vs. no (*n* = 37)	1.609	0.773–3.353	*0.204*	1.429	0.581–3.516	*0.437*
Resection of primary tumour (*n* = 23) vs. no resection (*n* = 27)	0.771	0.386–1.543	*0.463*	0.614	0.279–1.352	*0.226*
Bone metastases Yes (*n* = 19) vs. no (*n* = 31)	2.710	1.285–5.716	***0.009***	2.637	1.205–5.772	***0.015***

All *p*-values have been specified as italicized values and all values showing statistical significance were highlighted using bold characters

^a^Everolimus and/ or sunitinib

### Toxicity

Adverse effects were documented in 49 of the 50 patients. The most common side effect reported was constipation in 28 patients (57%). This could be controlled with laxatives and enemas without restricting the activities of daily living (ADL). Fatigue occurred in 11 patients (22%) without limiting the ADL. Nausea and vomiting occurred in eight patients (16%) and were mild to moderate. In two patients (4%), hospitalisation was prolonged by persistent nausea despite adequate therapy. At the onset of chemotherapy, 19 patients (39%) had anaemia with haemoglobin (Hb) levels between the specific lower limit of normal and 10 g/dl; eight patients (16%) had anaemia with Hb values between 10 and 8 g/dl. A decrease in the initial Hb value, corresponding to CTCAE grade 1, was found in 11 patients (22%) during STZ CTx. No patient showed a Hb-level decrease of grade 2 or higher. In two patients (4%), leukopenia occurred, one case of leukopenia led to discontinuation of STZ CTx after four cycles owing to persistent fever. Eight patients had pre-therapeutic thrombocytopenia. In two patients (4%), thrombocytopenia first appeared on therapy (CTCAE grade 2). Liver enzymes (aspartate aminotransferase, alanine aminotransferase and gamma-glutamyltransferase) increased in 21 patients (43%) while on therapy, corresponding to CTCAE grades 1 and 2. Renal function could be assessed in 48 patients using creatinine levels and glomerular filtration rate (GFR) calculated according to the Modification of Diet in Renal Disease (MDRD) formula. Prior to STZ CTx, 41 patients had normal renal function and seven patients had a slightly reduced function. Chemotherapy resulted in a mostly mild deterioration of renal function in 13 patients (27%) (Table 4). Additionally, decreased renal function parameters 6 and 12 months after cessation of chemotherapy were reported in 35 and 27 patients, respectively. GFR decreased in the first 6 months after cessation of STZ CTx and then remained stable (Table 5). No treatment-related deaths were observed.

**Table 4:** Toxic reaction to chemotherapy

Reaction	All grades No. (%)	Grade 3 or 4 No. (%)
Constipation	28 (56)	0
Altered liver enzymes	21 (43)	2 (4)
Renal insufficiency	13 (27)	1 (2)
Anaemia	11 (22)	0
Fatigue	11 (22)	0
Nausea/vomiting	8 (16)	2 (4)
Leukopenia	2 (4)	1 (2)
Thrombocytopenia	2 (4)	0

**Table 5:** Renal function (GFR^a^, MDRD^b^ formula) before and after STZ CTx

	Start STZ CTx *n* (%)^c^	Stop STZ CTx *n* (%)^c^	6 months after the end of STZ CTx *n* (%)^d^	12 months after the end of STZ CTx *n* (%)^e^
>60 ml/min	41 (85)	35 (73)	20 (57)	14 (52)
60–30 ml/min	7 (15)	12 (25)	14 (40)	11 (41)
29–15 ml/min	0 (0)	1 (2)	1 (3)	2 (7)
<15 ml/min	0	0	0	0

^*a*^*GFR* glomerular filtration rate.

^*b*^*MDRD* Modification of Diet in Renal Disease.

^*c*^*GFR* available in 48 patients.

^*d*^*GFR* available in 35 patients.

^*e*^*GFR* available in 27 patients.

## Discussion

In this study, the efficacy of STZ CTx in patients selected according to current guidelines was investigated [13, 20, 21]. Since the introduction of STZ CTx in the 1970s, the therapy threshold has risen from the mere detection of residual disease to the clinical or radiological progression of an incurable condition [3, 13, 22]. In 2010, competing targeted therapies with sunitinib and everolimus were approved and PRRT became widely available [7, 8, 11]. Since then, STZ CTx has been used competitively with these new therapies, preferably in short-term progressive PanNET with multiple-organ manifestations. This study indicates a persistent ORR of 38% with a lower PFS at 12 months and lower OS of 38 months compared with previous studies (Table 6).

**Table 6:** Patient characteristics and outcome of STZ CTx in PanNET

	Regimen, implementation (duration)	*n*	Radiologic response assessable, *n* (%)	Localisation	WHO tumour grading G1 (% of cohort)	Stage IV	STZ CTx 1^st^ line	Time from initial diagnosis to therapy start (months)	Progressive disease at baseline	ORR	DCR	mPFS/TTP (months)	mOS (months)	Markers of response	Prognostic factors PFS/TTP	Prognostic factors OS
Clewemar et al. [15]	STZ/5-FU 1981–2014	133	100 (75.2%)	100% pancreas	36%	88%	63.2%	n.r^a^	32%	28%	92%	23	51.9	CgA decrease > 50%	Grading Stage IV	Grading Previous surgery
Dilz et al. [13]	STZ/5-FU 1998–2014	96	93 (96.8%)	100% pancreas	12%	93.8%	56.3%	11.8	74%	42.7%	83.3%	19.4	54.8	CgA decrease >30%	Ki-67 > 15%	Ki-67 > 15% Metastatic sites ≥2
Krug et al. [14]	STZ/Dox/5-FU 1995–2013	77	64 (83.1%)	84.4% pancreas (+bronchial, duodenal, CUP-NEN)	12%	90.9%	19.5%	33	n.r^a^	34%	72%	16	28	CgA decrease >30%, positive Octreo-Scan	Ki-67 > 10%	—^b^
Schrader et al. [16]	STZ/5-FU 2002–2018	30	28 (93.3%)	100% pancreas	n.r^a^	92.9%	61%	n.r^a^	n.r^a^	36%	86%	21	69	—^b^	Previous surgery	—^b^
Lahner et al.	STZ/5-FU 2010–2018	50	50 (100%)	100% pancreas	6%	96%	54%	6.5	100%	38%	76%	12	38	CgA decrease >30%	—^b^	Bone metastases Therapy line

^*a*^*n.r*. not reported

^*b*^*—* not found

Baseline characteristics of our cohort confirmed a shift toward a more aggressive clinical course compared with published data [14–17]. The median duration of illness from the first diagnosis to the beginning of STZ CTx was considerably shorter in our investigation (6.5 months) than in recent studies from Berlin and Marburg (11.8 and 33.0 months, respectively) [15, 16]. At the same time, 50% of our patients had a tumour spread to two or more distant organ systems; in a previous study, tumours spread in only 33% of patients [15]. The proportion of patients with a low proliferative G1 PanNET, corresponding to a Ki-67 index of ≤2%, was significantly smaller in our cohort (6%) compared with that in previous studies, with G1 PanNET of 12%–36% [14–16]. Finally, when commencing STZ CTx, all of our patients were morphologically and/or clinically progressive. Taken together, our cohort illustrates the influence of the guidelines, treating preferably short-term progressive PanNET G2 or G3 with multiple-organ manifestations.

Despite this selection, we observed an ORR in 38% of the patients, which is in line with the results of 34%, 36%, and 42.7% in previous studies [15–17]. In contrast, 24% of our patients progressed during therapy, corresponding to a DCR of 76%, which is lower than previous reports [14–17]. Interestingly, the highest DCR of STZ CTx (92%) was found in a study that had a uniquely high proportion of low proliferative G1 PanNET patients (36%), possibly reflecting the natural course of the disease rather than anti-tumour activity [14]. With a decreasing proportion of G1 differentiated PanNET, the DCR also decreased [15, 17]. Even progression status at baseline may play a relevant role. In previous reports focused on STZ CTx, progression status was rarely reported. However, in two prospective, placebo-controlled PanNET studies with sunitinib and everolimus in progressive patients, the DCRs of 72% and 73% match the 76% DCR of our STZ CTx study [7, 8]. Compared with targeted therapy, our results show a superior ORR with a similar DCR. As shown before, the biochemical response (CgA decrease >30%) was associated with significantly higher ORR. However, while there was a trend toward better response of STZ–CTx without preceding therapy with targeted therapy or PRRT, this difference was not statistically significant as the sample size might lack the power to reliably detect a difference.

The median PFS of 12 months and median OS of 38 months were considerably shorter in our cohort than in recent analyses. Clewemar et al. reported a much higher PFS of 23 months with an OS of 51.9 months in patients treated between 1981 and 2014 [14]. Dilz et al. showed a time to progression (TTP) of 19.4 months with an OS of 54.8 months in patients treated between 1998 and 2014 [15]. Krug et al. and Schrader et al. reported similar results; the OS was exceptionally short in the first study, most likely because of the inclusion of bronchial and CUP-NET [16, 17] (Table 6). PFS and OS in our study reflected the performance of STZ CTx in progressive PanNET patients with multiple-organ manifestations.

Previous lines of therapy, including surgery, had no measurable impact on PFS. However, data on targeted therapy were limited to seven patients and on PRRT to 13 patients. The impact of prior treatments, therefore, needs to be further elucidated in a larger cohort.

Interestingly, we could not find any influence of the Ki-67 marker on PFS, as described in other studies. However, only three of our patients (6% of the cohort) had a WHO G1 tumour; thus, a much smaller proportion of our patients had G1 tumours than that in the other studies (Table 6). Again, this demonstrates the influence of patient selection according to the current guidelines from 2010 onwards. The G1 PanNET patients whose biologically slow course ultimately shows the influence of the Ki-67 marker are only rarely treated with STZ CTx nowadays.

STZ CTx as the first-line therapy was associated with a significantly longer median OS in this study. Thus, beginning the therapy sequence with STZ CTx may result in a more favourable outcome for dynamically growing PanNET. On the other hand, available therapy alternatives may have an impact. Everolimus, sunitinib, PRRT, or temozolomide/capecitabine may be administered in subsequent lines of therapy to our patients. First-line therapy may be associated with the longest survival in this setting. In contrast, no reliable therapy alternatives were available for patients in past decades.

Another key finding in this study is the prognostic importance of bone metastases. In this study, 38% of our patients had bone metastases, which was significantly higher than would have been expected. An analysis of 14,685 GI-NEN patients in the Surveillance, Epidemiology, and End Results database (SEER-9 registry) from 1973 to 2015 showed a bone metastases rate of 5.7% in stage-IV patients [23]. In contrast, the rate of bone metastases in an analysis by a tertiary referral centre in Germany was 26% in stage-IV patients [24]. Notably, a significant increase in bone metastases was detected after the introduction of 68Ga-DOTATOC PET/CT. In our study, all patients had received hybrid imaging before beginning STZ CTx and at least once a year thereafter, so the increased incidence of bone metastases in our study may have been due to improved detection corresponding to true incidence. In addition, the selection of patients with a more aggressive course may have also impacted the bone metastasis rate. Overall, the significant influence of bone metastases on the median OS is remarkable; the OS was reduced to nearly half in patients with bone metastases compared with that in patients without bone metastases (24 vs. 46 months) (Fig. 4).

In contrast to previous studies, we detected no effects of primary tumour resection, the Ki-67 index, or the number of metastatic sites on median OS [14, 15]. Surgical removal of the primary tumour, as a prognostic factor, is prone to selection bias, because patients with a smaller tumour burden, lower grading and better performance status may be more likely to undergo surgery. A higher Ki-67 index and more affected organs, which were previously mentioned as prognostic parameters, are statistically included in our collective as an initial finding and, therefore, are no longer detectable. Interestingly, neither preceding targeted therapy nor PRRT showed a significant correlation with median PFS or OS. It should be noted that the number of patients with targeted therapy prior to STZ CTx was small in our analysis. The ongoing phase-3 SEQTOR study (NCT 02246127), which compares the STZ CTx followed by everolimus upon progression with the reverse sequence, will further elucidate the optimal therapy sequencing.

The safety profile of STZ CTx in our study was consistent with the previous experience in advanced PanNET. The most frequent toxic reactions were of grade 1 or 2 severity and included renal insufficiency, anaemia, fatigue, and nausea; the frequencies were similar to those reported previously [14–16].

## Conclusion

The majority of patients with dynamically progressive PanNET benefit from STZ CTx. The toxicity is low. Compared with those in targeted therapies or PRRT, remissions occur more frequently. First-line STZ CTx is associated with prolonged survival. Patients with bone metastases require intensive therapy.
